# Anticoagulant activity of apixaban can be estimated by multiple regression analysis

**DOI:** 10.1002/joa3.12435

**Published:** 2020-09-22

**Authors:** Naoko Unami, Yuya Ise, Hidenori Suzuki

**Affiliations:** ^1^ Department of Pharmaceutical Services Nippon Medical School Hospital Bunkyo‐ku Japan; ^2^ Department of Pharmacology Graduate School of Medicine Nippon Medical School Bunkyo‐ku Japan

**Keywords:** anticoagulants, apixaban, atrial fibrillation, DOAC, prothrombin time

## Abstract

**Background:**

Information on apixaban anticoagulant activity is required to prevent major bleeding or thrombosis during its use.

**Methods:**

We enrolled 194 patients with nonvalvular atrial fibrillation (NVAF) in whom warfarin was replaced with apixaban: 105 (54.1%) received the standard dose of apixaban (5 mg twice daily [BID]; 5 mg group) and 89 (45.9%) received a reduced dose (2.5 mg BID; 2.5 mg group). Multiple regression analysis was performed to predict the prothrombin time of apixaban (PTa) based on factors including age, body weight (BW), serum creatinine, and CHA_2_DS_2_‐VASc score.

**Results:**

PTa and PT of warfarin (PTw) were significantly correlated in both groups (correlation coefficient R = 0.239 [*P* = .014] in the 5 mg group; R = 0.248 [*P* = .019] in the 2.5 mg group). PTa in the 5 mg group was predicted as follows: 16.952‐0.036 × BW +0.299 × CHA_2_DS_2_‐VASc score (*P* < .0004; R = 0.378). However, in the 2.5 mg group, PTa could not be predicted. The mean of the predicted and measured PTa values in the 5 mg group was 15.6 s, which was similar to the mean measured PTa of 15.5 s in the 2.5 mg group.

**Conclusions:**

PT can be predicted by a formula including simple clinical parameters in patients receiving the standard dose of apixaban. This simple predictive formula may help to stratify bleeding and thrombosis risks in patients treated with apixaban.

## Graphical abstract

Estimation of anticoagulant activity of apixaban.

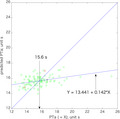

## 1 INTRODUCTION

An epidemiological study of atrial fibrillation (AF) indicated that its prevalence tended to increase with age.^1^ Kodani et al reported that the incidence rate of new‐onset AF was 2.5/1000 person‐years.^2^ Patients with nonvalvular AF (NVAF) have an average incidence of cerebral infarction of 5% per year.^3^, ^4^, ^5^

Although warfarin was the only oral anticoagulant used to prevent cerebral infarction in patients with NVAF for many years, it has been replaced within the last 10 years by direct oral anticoagulants (DOACs), such as dabigatran, rivaroxaban, edoxaban, and apixaban. The rapid increase in the use of DOACs has occurred because they do not require frequent monitoring of anticoagulant activity or dose adjustment, and their anticoagulant activity is greater than or equal to that of warfarin.^6^ Apixaban was first approved in the European Union, Canada, the United States, and Japan in 2012, and several cohort studies have recently reported that apixaban was associated with a lower risk of major bleeding compared with warfarin.^7^, ^8^, ^9^ However, the more widespread use of DOACs has revealed various complications, such as bleeding or thrombosis, and measurement of their anticoagulant activity and/or plasma levels under these circumstances may be useful. A readily available method for determining the anticoagulant activity of DOACs is therefore necessary to prevent bleeding in patients receiving these drugs in a preoperative or emergency setting. Anti‐factor Xa (FXa) chromogenic assays are considered to be the most appropriate assays for the direct quantitative measurement of FXa inhibitor plasma levels.^10^ Moreover, the need for agents to neutralize DOACs and prevent anticipated bleeding during waiting periods and emergency operations has also increased. Idarucizumab was approved as a specific neutralizer for dabigatran in Japan in November 2016, and andexanet alfa has been developed as a specific neutralizer for apixaban and rivaroxaban.^11^, ^12^ Although routine monitoring of anticoagulant activity and dose adjustments is not required with DOACs, the above findings indicate the need for more information on the anticoagulant activity of DOACs.

The anticoagulation mechanisms of DOACs must be considered in order to measure their anticoagulant activity. Activated partial thromboplastin time (APTT) is considered to be a sensitive test for assaying the direct thrombin (IIa) inhibitor, dabigatran.^13^ Rivaroxaban, edoxaban, and apixaban are direct FXa inhibitors, and prothrombin time (PT) can therefore be used to monitor their concentrations in plasma.^14^, ^15^ Shinplastin Excel S^®^ (Kyowa Medex Co, Tokyo, Japan) and Coagpia PT‐N^®^ (Sekisui Medical Co, Tokyo, Japan) have been used as specific reagents to predict the anticoagulant activity of apixaban from PT.^16^ Shinplastin Excel S^®^ and Coagpia PT‐N^®^ are tissue thromboplastin reagents derived from human brain and rabbit brain, respectively. Thrombocheck PT Plus^®^ (Sysmex Co, Kobe, Japan), which uses the same tissue thromboplastin reagent (rabbit brain) as Coagpia PT‐N, has also been used at Nippon Medical School Hospital. We conducted a preliminary study to determine if PT was significantly prolonged by the administration of apixaban.^17^ Twenty‐three patients with NVAF received apixaban (5 mg tablets twice daily [BID]), and the PT was significantly prolonged by apixaban (PTa) compared with the normal PT range of 10‐12 s (*P* = .01).

In the current study, we investigated the use of PTa as a measure of anticoagulant activity in patients taking apixaban 2.5 mg BID or 5 mg BID. We also determined if PTa could be predicted by patient‐specific variables based on multiple regression analysis, and if a standard PT value could be identified to reflect the anticoagulant activity of apixaban.

## 2 METHODS

### 2.1 Subjects

The present study was conducted at Nippon Medical School Hospital. Warfarin was replaced with apixaban (5 mg or 2.5 mg BID) in 441 patients with NVAF from February 1, 2013 to July 31, 2017. A reduced dose of 2.5 mg BID was recommended if the patient met at least two of the following conditions: age ≥80 years, body weight ≤60 kg, and serum creatinine >1.5 mg/dL.^18^ These subjects did not satisfy the exclusion criteria for the ARISTOTLE trial (Apixaban for Reduction in Stroke and Other Thromboembolic Events in Atrial Fibrillation), which were as follows: AF of a reversible origin, moderate to severe mitral stenosis, requirement of anticoagulant therapy for an artificial cardiac valve, stroke occurring within 7 days, concurrent administration of aspirin >165 mg/day or aspirin + clopidogrel, and severe kidney dysfunction (serum creatinine >2.5 mg/dL or estimated creatinine clearance <25 mL/min).^19^ Among the 441 patients, 200 subjects met the inclusion criteria for the current study: PT of warfarin (PTw) measured within 30 days before replacing warfarin with apixaban, and PTa measured at least once within 1‐4 weeks after the replacement medication. Because the duration of warfarin action is about 72 h, warfarin did not affect the measurement of PTa. Drugs considered to affect blood levels of apixaban were listed on the package insert for apixaban.^18^ Among these, diltiazem has been reported to increase the blood levels of apixaban, and six patients receiving diltiazem were therefore excluded from the study. The remaining 194 patients received no such medicines and were therefore included in the final analysis. Among these 194 recruited patients, 105 (54.1%) received the standard dose of apixaban (5 mg BID; 5 mg group), and 89 (45.9%) received a reduced dose (2.5 mg BID; 2.5 mg group). Because the study was retrospective, the name and identification number of each subject were replaced with a reference number, which met the hospital's ethics criteria. The protocol was approved by the Ethics Committee of Nippon Medical School Hospital for compliance with ethical standards.

Acute major bleeding was defined as bleeding meeting at least one of the following criteria: potentially life‐threatening bleeding, bleeding causing a decrease in hemoglobin level ≥2 g/dL, or bleeding in a critical area (eg, intracranial and pericardial).^12^ No subjects had acute major bleeding while taking apixaban. Regarding minor bleeding, one patient in the 5 mg group experienced recurrent epistaxis and one patient in the 2.5 mg group developed subcutaneous bleeding while taking apixaban. Apixaban was replaced with warfarin and dabigatran, respectively, in these two patients. Cerebral infarction occurred in one patient in the 5 mg group, and apixaban was changed to dabigatran in one patient in the 5 mg group because of a lack of effect on a thrombus in the left appendage.

### 2.2 Evaluation

The medical records of patients were reviewed retrospectively to collect information on sex (male/female), age (years), height (cm), body weight (kg), dose of warfarin (mg/day), apixaban dose (5 or 2.5 mg BID), serum creatinine (mg/dL), prothrombin time‐international normalized ratio (PTINR) when taking warfarin, PTINR when taking apixaban, PT (s) when taking warfarin (PTw), PT (s) when taking apixaban (PTa), CHA_2_DS_2_‐VASc score (points), and CHADS_2_ score (points). CHA_2_DS_2_‐VASc score was calculated as follows: 1 point each for congestive heart failure, hypertension, age 64‐74 years, diabetes mellitus, vascular disease (previous myocardial infarction, peripheral artery disease, or aortic plaque), and female sex, and 2 points each for age ≥75 years and stroke/transient ischemic attack (TIA)/thromboembolic disease.^20^ The CHADS_2_ score was calculated as follows: 1 point each for congestive heart failure, hypertension, age ≥75 years, and diabetes mellitus, and 2 points each for stroke/TIA/thromboembolic disease.^21^ Body surface area was calculated using DuBois's Equation. ^22^ Estimated creatinine clearance was calculated using the Cockcroft‐Gault Equation. ^23^

Blood was collected at various times after oral administration of apixaban. PT was measured using automated coagulation analyzers, including COAPRESTA2000^®^ (CP2000) until December 2016 and CP3000 after January 2017. Thrombocheck PT Plus^®^ was used until April 2018, and Coagpia PT‐N^®^ (which used the same tissue thromboplastin reagent [rabbit brain] as Thrombocheck PT Plus^®^) was used after May 2018. Although PT generally varies slightly depending on the measuring equipment and reagents, it was adjusted to an error of <0.1 s using a calibration lot.

### 2.3 Statistical analysis

Data are expressed as mean ± standard deviation. Student's unpaired t‐test was used to compare results between the 5 mg group and the 2.5 mg group. The numbers of males and females were compared by χ^2^ test. If PT was measured more than once while taking apixaban, the maximum PT was used as PTa. Because PTa was considered to be prolonged based on the mechanism of apixaban, the significance of the prolongation compared with the normal range (10‐12 s) was examined using a one‐sided test. PTw was compared with PTa using Student's paired *t* test.

Multiple regression analysis was performed to predict PTa from clinical variables. We determined how many variables could be used in the multiple regression analysis based on the anticipated effect size, desired statistical power level, probability level, and the sample size. Following convention, the anticipated effect size was set at 0.15, the desired statistical power level was set at 0.8, and the probability level was set at 0.05.^24^ The number of subjects in the 5 mg group was 105 and the number in the 2.5 mg group was 89, and the maximum number of explanatory variables was therefore calculated to be four for a sample size of 89.^24^ When selecting explanatory variables for multiple regression analysis, care must be taken to avoid multicollinearity: some combinations of variables may be highly correlated with each other, in which case there is no need to include both as possible explanatory variables. We therefore selected the possible explanatory variables accordingly. StatView ver.5.0 (SAS Institute Inc, Cary, NC, USA) was used for the statistical analyses. Statistical significance was considered at *P* ≤ .05 in all cases.

## 3 RESULTS

The subjects’ clinical characteristics are listed in Table 1. There were significant differences between the 5 mg group and the 2.5 mg group in terms of sex ratio, age, body weight, dose of warfarin, serum creatinine, creatinine clearance, CHA_2_DS_2_‐VASc score, and CHADS_2_ score. There was no significant difference in PTw or PTa between the 5 mg group and the 2.5 mg group.

**Table 1:** Patient characteristics

Dose	5 mg BID (n = 105)	2.5 mg BID (n = 89)	*P* value
Age (years)	70.8 ± 8.8	80.5 ± 6.6	*P* < .0001*
Sex (male/female)	29/76	47/42	*P* < .001**
Body weight (kg)	62.4 ± 12.5	52.4 ± 12.3	*P* < .0001*
Dose of warfarin (mg/day)	2.8 ± 1.2	2.3 ± 1.1	*P* = .0024*
Serum creatinine (mg/dL)	0.9 ± 0.2	1.0 ± 0.3	*P* = .0106*
Creatinine clearance (mL/min)	61.3 ± 19.3	42.8 ± 15.0	*P* < .0001*
PT while taking warfarin (s)	20.0 ± 5.5	20.3 ± 6.6	*P* = .6869
PT while taking apixaban (s)	15.6 ± 1.8	15.5 ± 1.8	*P* = .5948
CHA_2_DS_2_‐VASc (points)	3.2 ± 1.5	4.3 ± 1.5	*P* < .0001*
CHADS_2_ (points)	2.0 ± 1.3	2.8 ± 1.3	*P* < .0001*

Data expressed as mean ± standard deviation. PT, prothrombin time.

* Compared by Student's *t* test.

** Compared by χ^2^ test.

**Figure 1:**
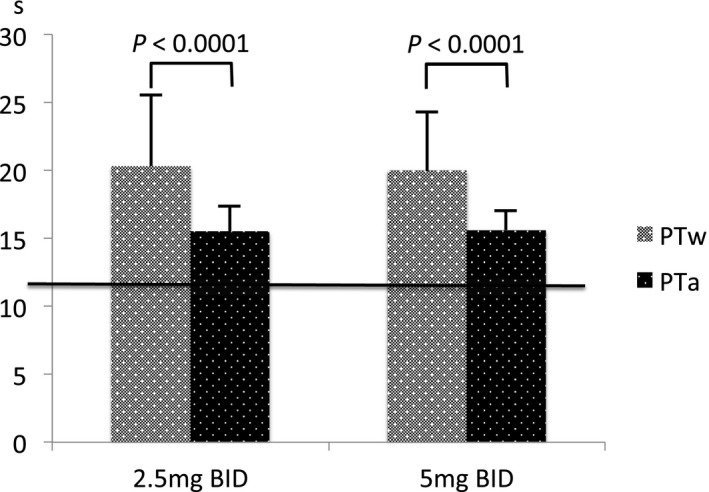
Student's paired t‐test was used to compare prothrombin time while taking warfarin (PTw) and prothrombin time while taking apixaban (PTa). The horizontal line at 12 s shows the upper limit of the normal PT range

PTw was significantly longer than PTa in both groups (paired Student's *t* test; *P* < .0001, Figure 1). PTa values in both groups (Table 1) were significantly prolonged compared with the normal range of 10‐12 s (*P* ≤ .05). The correlation between PTa and PTw was significant in both groups (correlation coefficient R = 0.239 [*P* = .014] in the 5 mg group; R = 0.248 [*P* = .019] in the 2.5 mg group).

Considering the number of subjects, we included a maximum of four predictors in the multiple regression analysis. Age, body weight, serum creatinine (or creatinine clearance), and CHA_2_DS_2_‐VASc score (or CHADS_2_ score) were chosen as the candidate predictors. In the event of a linear relationship between any of these explanatory variables, only one variable must be selected to avoid multicollinearity. Regarding the better candidate for multiple regression analysis between the CHA_2_DS_2_‐VASc and CHADS_2_ scores, the correlation between PTa and CHA_2_DS_2_‐VASc score in the 5 mg group was significant (*P* = .0033; correlation coefficient R = 0.283) while the correlation between PTa and CHADS_2_ score was not significant (*P* = .0935; R = 0.165), suggesting that CHA_2_DS_2_‐VASc score might be a better predictive factor than CHADS_2_ score. The correlations between PTa and serum creatinine and between PTa and creatinine clearance were not significant (*P* = .3375; *P* = .2537, respectively). Because creatinine clearance is based on serum creatinine, body weight, age, and sex, body weight and age may have been collinear with creatinine clearance, and we therefore selected serum creatinine as the potential predictive factor for PTa. Neither the correlation between PTa and CHA_2_DS_2_‐VASc score nor between PTa and CHADS_2_ score was significant in the 2.5 mg group (*P* = .2947; *P* = .3457, respectively), and neither the correlation between PTa and serum creatinine nor between PTa and creatinine clearance was significant (*P* = .8652; *P* = .9090, respectively). Multiple regression analysis was therefore performed in both the 5 mg group and the 2.5 mg group using age, body weight, serum creatinine, and CHA_2_DS_2_‐VASc score as tentative predictors of PTa (Table 2). Body weight was a significant predictor of PTa and CHA_2_DS_2_‐VASc score was nearly significant in the 5 mg group, but none of the variables were significant predictors of PTa in the 2.5 mg group. Regression analysis was performed in the 5 mg group using two different sets of explanatory variables: body weight and CHA_2_DS_2_‐VASc score, and body weight (Table 3). The correlation was larger in the former compared with the latter. In Table 3, the T value indicates the magnitude of the effect of each predictor on PTa (larger absolute value, stronger effect). Body weight and CHA_2_DS_2_‐VASc score both affected PTa in the 5 mg group (absolute T values > 2; Table 3) and these were therefore identified as predictors of PTa. However, there were no significant predictors of PTa in the 2.5 mg group (Table 2), as confirmed by multiple regression analysis (Table 3).

**Table 2:** Predictors of PTa

	*P* value	*P* value
Predictors	(5 mg BID; n = 105)	(2.5 mg BID; n = 89)
Age	.2859	.9052
Body weight	.0165*	.7392
Serum creatinine	.8355	.8178
CHA_2_DS_2_‐VASc	.0876	.2606

Abbreviation: PTa, prothrombin time in patients taking apixaban.

**Table 3:** Multiple regression analysis to predict PTa

	Co; T value; *P* value	Co; T value; *P* value	Co; T value; *P* value
Predictors	A (5 mg BID; n = 105)	B (5 mg BID; n = 105)	(2.5 mg BID; n = 89)
BW	‐0.036; −2.725; 0.0076*	‐0.042; −3.076; 0.0027*	0.007; 0.467; 0.6415
CHA2DS2‐VASc	0.299; 2.641; 0.0096*	none	0.155; 1.142; 0.2566
	*P* < .0004* (R = 0.378)	*P* = .0027* (R = 0.290)	*P* = .5185 (R = 0.123)

Abbreviations: BW, body weight; Co, coefficient; PTa, prothrombin time in patients taking apixaban; R, correlation coefficient.

* Statistically significant.

**Figure 2:**
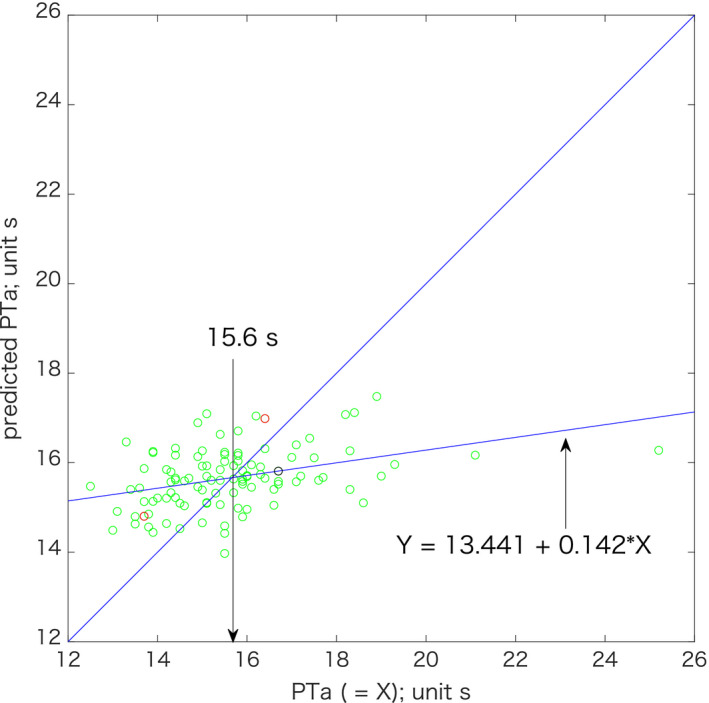
Scatter plot of measured prothrombin time in patients taking 5 mg apixaban twice daily (BID) (x axis) compared with predicted PTa (y axis) using multiple regression analysis. Two red circles above the diagonal represent a patient with cerebral infarction (right circle) and a patient with thrombus in the left appendage (left circle), respectively. The black circle below the diagonal represents a patient with recurrent epistaxis. The predicted line and the diagonal line intersect at 15.6 s

PTa in the 5 mg group was predicted by the following formula:

predicted PTa=16.952‐0.036×BW+0.299×CHA2DS2‐VASc score

where BW is the body weight (*P* < .0004; R = 0.378; Table 3). Because R = 0.378 (≈0.4), the measured PTa and predicted PTa were moderately correlated. PTa was not significantly correlated with CHA_2_DS_2_‐VASc score (*P* = .0876) in multiple regression analysis using age, body weight, serum creatinine, and CHA_2_DS_2_‐VASc score as tentative predictors of PTa (Table 2); however, CHA_2_DS_2_‐VASc score significantly contributed to the prediction of PTa according to multiple regression analysis using body weight and CHA_2_DS_2_‐VASc score (Table 3). Using this formula, the anticoagulant activity of 5 mg apixaban BID can be predicted. The mean predicted PTa of 15.6 s was the same as the mean measured PTa (Table 1). Points above the diagonal in Figure 2 indicate that the measured PT was lower than the predicted PT, suggesting that the anticoagulant activity may be insufficient. Two red circles above the diagonal in Figure 2 were from a patient with cerebral infarction and a patient with thrombus in the left appendage, respectively. Conversely, circles below the diagonal indicated that the measured PT was higher than the predicted PT, suggesting that the anticoagulant activity may be over‐effective. One black circle below the diagonal was from a patient with recurrent epistaxis.

Simple regression analysis using only body weight resulted in a lower correlation coefficient and higher P value compared with multiple regression analysis using body weight and CHA_2_DS_2_‐VASc score (Table 3).

## 4 DISCUSSION

Apixaban has very clear criteria for administering a reduced dose: age ≥80 years, serum creatinine ≥1.5 mg/dL, and body weight ≤60 kg. It is therefore acknowledged that this drug can be administered at a safe dose to prevent the side effect of bleeding, which is the most notable adverse feature of anticoagulants. Although the incidence of major bleeding with apixaban was lower compared with warfarin (2.13% vs 3.09%), among the 18,201 patients in the ARISTOTLE trial,^25^ it might still be necessary to monitor the anticoagulant activity of apixaban, as well as warfarin, to prevent major bleeding, especially during emergency surgery or traumatic hemorrhage. Warfarin requires frequent monitoring of anticoagulant activity or dose adjustments, and DOACs were therefore developed to avoid such routines. The anti‐FXa assay was reported to be reliable, because anti‐FXa activity is the preferred indicator of apixaban plasma concentrations;^10^ however, this assay is not widely used. Inoue et al recently reported higher incidence rates of thromboembolic and major hemorrhagic events in patients treated with 2.5 mg compared with 5 mg apixaban.^26^ We therefore sought to develop a simple method of measuring the anticoagulant activity of apixaban, and determined if it could be estimated from PT and/or other clinical parameters.

PTa was initially unavailable because of the small peak‐trough variability and poor sensitivity for predicting anticoagulant activity.^14^ However, PT was reported to be prolonged using Triniclot PT Excel S^®^ (Japanese trade name Shinplastin Excel S^®^),^27^ and subsequently using Coagpia PT‐N^®^.^16^ Because Thrombocheck PT Plus^®^ uses the same tissue thromboplastin reagent as Coagpia PT‐ N^®^, we therefore presumed that it could be used to estimate apixaban‐associated FXa activity in the same way as in Kanemoto et al.^16^ Thrombocheck PT Plus^®^ and Coagpia PT‐N^®^ were used in our hospital. PTa was significantly shorter than PTw, suggesting that the sensitivity of PT to the anticoagulant activity of apixaban was lower than that to warfarin. However, the significant prolongation of PTa over the normal range in the present study was presumed to reflect the anticoagulant activity of apixaban. Although the anticoagulant mechanisms of apixaban and warfarin differ, the weak but significant correlation between PTa and PTw indicated that PTa reflected the anticoagulant activity, thus confirming that PTa could be used as a measure of anticoagulant activity in patients taking apixaban 2.5 mg or 5 mg BID.

Age, body weight, dose of warfarin, serum creatinine, creatinine clearance, CHA_2_DS_2_‐VASc score, and CHADS_2_ score differed significantly between the 5 mg and 2.5 mg groups. This was probably related to the dose reduction criteria, which recommend a reduced dose of 2.5 mg BID in patients with NVAF with two or more of the following characteristics: age ≥80 years, serum creatinine ≥1.5 mg/dL, and body weight ≤60 kg. The ratio of women to men was significantly higher in the 5 mg group compared with the 2.5 mg group, but the reverse was reported in other studies.^26^, ^28^, ^29^ This may have been caused by the relatively small sample size in the present study (194 patients) compared with the other studies (>5500 patients).

Age, body weight, serum creatinine (or creatinine clearance), and CHA_2_DS_2_‐VASc score (or CHADS_2_ score) were listed as candidate predictors. Examination of the correlations with PTa revealed that neither serum creatinine nor creatinine clearance was a significant predictor. This was thought to be because apixaban has several different metabolic pathways and is less dependent on renal function.^15^

PTa could be predicted using body weight and CHA_2_DS_2_‐VASc score in the 5 mg group (*P* < .0004, R = 0.378), and PT was thus considered to be an available index of the anticoagulant activity of apixaban. This confirmed that PTa could be predicted by patient‐specific variables based on multiple regression analysis. The negative coefficient of body weight in the PTa‐prediction formula indicates that the predicted PTa decreases as body weight increases. This may be because of the lower plasma apixaban concentration in patients with a higher body weight. This was consistent with the finding of Inoue et al, who showed that patients with lower body weight were more likely to develop a major hemorrhage.^26^ We presumed that the concentration of apixaban and the bleeding tendency would be increased in patients with a lower body weight. In the current study, the timing of PTa measurements after taking apixaban varied, and we presumed that the difference between the peak and trough plasma apixaban concentrations significantly affected the results. This could explain why the correlation between the measured and predicted PTa was at best moderate. We also considered that this explained why no significant predictive formula was obtained for the 2.5 mg group. The timing of blood sampling would thus affect the reference data (measured PTa), and this was not accounted for in the current study. This drawback should be taken into account when interpreting the results of this study.

The mean predicted and measured PTa values were the same (15.6 s) in the 5 mg group, suggesting that the mean measured PTa is a suitable standard value for apixaban anticoagulant activity. Moreover, this value was almost equal to the mean measured PTa (15.5 s) in the 2.5 mg group. Because of the similarities of these two values, 15.6 s was defined as the standard value for simplicity. If the PTa is much larger than this value, bleeding may occur, while if the PTa is much lower than this value, the anticoagulant effect may be insufficient. This confirmed that a standard PT value could be identified to reflect the anticoagulant activity of apixaban.

CHA_2_DS_2_‐VASc score is a categorical variable while the other variables are continuous variables. The correlation between PTa and predicted PTa was larger when PTa was predicted using body weight and CHA_2_DS_2_‐VASc score, compared with using body weight alone. CHA_2_DS_2_‐VASc score contributed to the prediction of PT because its T value was >2. However, because it is a categorical variable determined from multiple factors, it is not clear how CHA_2_DS_2_‐VASc score relates to the prediction.

There was no significant difference in PTa between the 5 mg group and the 2.5 mg group in the present study, suggesting no significant difference in anticoagulant activity between the groups. It is presumed that the three conditions for apixaban dose reduction (age ≥80 years, serum creatinine ≥1.5 mg/dL, and body weight ≤60 kg) resulted in equivalent blood levels to the standard dose, despite the dose reduction.

The present study was an observational study conducted at a single hospital, and the total number of patients receiving apixaban was therefore small compared with other studies on apixaban safety and efficacy.^26^, ^28^, ^29^ Additionally, the number of patients was further reduced by being limited to patients who were switched from warfarin to apixaban. However, the number of patients was sufficient to allow up to four possible explanatory variables to be included in the multiple regression analysis. The correlation coefficient between the predicted and the measured PT (PTa) was only moderate at best in the 5 mg group, and the prediction accuracy of PT during apixaban administration was not high. This may have been because the timing of blood sampling would influence the reference data (measured PTa), and this was not taken into account in the current study.

## 5 CONCLUSIONS

We concluded that PT reflects the anticoagulant activity of apixaban. The PT value in patients receiving the standard dose of apixaban can be predicted by a formula using simple clinical parameters (body weight and CHA_2_DS_2_‐VASc score). This simple predictive formula may help to stratify bleeding and thrombosis risks in patients treated with apixaban.

## CONFLICT OF INTEREST

The authors declare no conflict of interests for this article.

## ETHICAL APPROVAL

The protocol for this research project was approved by the Ethics Committee of Nippon Medical School Hospital (IRB approval number 29‐04‐752; June 23, 2017) and conforms to the provisions of the Declaration of Helsinki.
